# Postponed effect of neostigmine on oxidative homeostasis

**DOI:** 10.2478/intox-2014-0018

**Published:** 2014-12-30

**Authors:** Miroslav Pohanka

**Affiliations:** 1 Faculty of Military Health Sciences, University of Defense, Hradec Kralove, Czech Republic; 2 Karel English College in Brno, Brno, Czech Republic

**Keywords:** acetylcholinesterase, butyrylcholinesterase, oxidative stress, antioxidant, excitotoxicity, glutathione, neostigmine, inhibitor

## Abstract

Cholinesterases are enzymes able to hydrolyze the neurotransmitter acetylcholine and thus to terminate transmission. Once the enzymes are inhibited, excitotoxicity can appear in the adjacent cells. It is well known that oxidative stress is involved in the toxicity of cholinesterase inhibitors. Commonly, stress follows inhibition of cholinesterases and disappears shortly afterwards. In the present experiment, it was decided to test the impact of an inhibitor, neostigmine, on oxidative stress in BALB/c mice after a longer interval. The animals were sacrificed three days after onset of the experiment and spleens and livers were collected. Reduced glutathione (GSH), glutathione reductase (GR), glutathione S-transferase (GST), thiobarbituric acid reactive substances (TBARS), ferric reducing antioxidant power (FRAP), caspase-3 and activity of acetylcholinesterase (AChE) were assayed. The tested markers were not altered with exceptions of FRAP. The FRAP values indicate accumulation of low molecular weight antioxidants in the examined organs. The role of low molecular weight antioxidants in the toxicity of AChE inhibitors is discussed.

## Introduction

Two cholinesterases occur in the body: AChE (EC 3.1.1.7.) and butyrylcholinesterase (BChE; EC 3.1.1.8.). The cholinesterases can be inhibited by a wide group of chemical compounds. From the chemical point of view, organophosphorus irreversible inhibitors, carbamate pseudo-irreversible inhibitors and disparate reversible inhibitors acting mainly via cation – π interactions are known (Pohanka, 2012a). On searching the literature, drugs, artificial toxins and natural toxins can be recognized between the inhibitors. Compounds inhibiting either AChE or both cholinesterases include *e.g.* Alzheimer disease drugs donepezil, galantamine, rivastigmine, huperzin, parasympathomimetic neostigmine and pyridostigmine, nerve agents sarin, soman, tabun, the pesticide carbofuran, and other toxins such as aflatoxins (Pohanka, 2011a, b).

In the body, inhibitors of cholinesterases stop hydrolysis of the neurotransmitter acetylcholine and thus they cause overstimulation of acetylcholine receptors (Marrs, 1993). Exposure to the inhibitors can lead to disparate effects as the inhibition can relate to nicotinic and muscarinic acetylcholine receptors in the central and peripheral nervous system (Klinkerberg *et al.*, 2011; Marrs, 1993; Pohanka, 2012a, b). Death can occur as a consequence of fatal poisoning with inhibitors of cholinesterases. Respiratory paralysis is a typical primary cause of death due to fatal poisoning (Cannard, 2006).

Oxidative stress is another complication following exposure to inhibitors of cholinesterases. Though there remain some controversies as to the importance of oxidative stress (Juranek & Bezek, 2005), oxidative imbalance starts early after poisoning with the inhibitors and is considered a serious pathological consequence (Milatovic *et al.*, 2006). The initiation of oxidative stress is based on excitotoxicity and metabolic depression (Chen, 2012). Antioxidant therapy can protect from the excitotoxicity induced pathology (Gupta *et al.*, 2001). Inhibitors of cholinesterases typically cause an oxidative insult lasting for hours (Milatovic *et al.*, 2006). However, extensive research on the postponed effects has not been done and it is not clear whether some imbalances or pathological consequences can remain. This paper is concerned with the postponed effect of exposure to a pseudo-irreversible inhibitor of both AChE and BChE: nesotigmine (3-([(dimethylamino)carbonyl]oxy)-*N,N,N*-trimethylbenzenaminium). Neostigmine was chosen as it does not easily cross the blood-brain barrier due to the presence of quaternary nitrogen moiety in the compound (Parisi & Francia, 2000). It serves here as a model compound easily available as a drug. The achieved conclusions can thus be defined more plausibly in the peripheral organs and a pertinent role of pathology related to the central nervous system can be neglected. The time interval of three days was chosen as a period long enough for resolution of oxidative stress and return into oxidative homeostasis.

## Material and methods

### Experiment on animals

The experiment was performed on two-month-old female BALB/c mice. The mice were purchased from BioTest (Konarovice, Czech Republic) and once received, they were kept in an air conditioned room (temperature 22±2 °C, humidity 50±10%, and light period from 7 a.m. to 7 p.m.). At the beginning of the experiment, the mice weighed 20±2 g and were in a good condition. The animals had no limits in access to food and water. The experiment was done in the vivarium of the Faculty of Military Health Sciences (Czech Republic) and it was permitted and supervised by the Ethical Committee of the Ministry of Defence, Czech Republic.

Neostigmine (received as salt neostigmine bromide in analytical purity from Sigma-Aldrich, St. Louis, MO, USA) was solved in saline in an amount to receive the below mentioned doses. The doses were derived from described experiments and known doses of neostigmine used for pharmacological purposes (Lederer *et al.*, 2010; Starec *et al.*, 1997). Solutions were applied in the amount of 100 µl subcutaneously into the hind limb. The total of 32 animals was divided into four groups. The first, controls, received 100 µl saline. The second group was exposed to 8.00 µg/kg, the third to 40.0 µg/kg, and the last to 200 µg/kg of neostigmine. After three days, the animals were sacrificed by cardiac puncture under CO_2_ anesthesia. The liver and spleen were sampled at –80 °C until *ex vivo* assays.

#### Ex vivo assays

The collected organ samples were milled by an Ultra-Turrax device (Ika Werke, Staufen, Germany). From the cortex region, 100 mg of the organ tested was cut, put into 1 ml phosphate buffered saline and milled by Ultra-Turrax for one minute.

AChE activity was assayed as follows: to 0.4 ml of 5,5′-dithiobis-(2-nitrobenzoic) acid 0.4 mg/ml and 100 µl tissue homogenate 400 µl of phosphate buffered saline was poured. The reaction was started by addition of acetylthiocholine chloride (100 µl; 10 mmol/l). Absorbance was measured at 412 nm five minutes after pouring and the enzyme activity was calculated using extinction coefficient ɛ=14,150 l/mol×cm. The principle of the assay is described in the papers by Ellman *et al.* (1961) and Pohanka (2013).

Caspase-3 activity was assayed using CASP3C kit (Sigma-Aldrich, St. Louis, MO, USA). The assay was processed in compliance with the protocol provided by the manufacturer. Standard 96 well microplates and multichannel spectrophotometer were used for the assay. FRAP, TBARS, GR, GST and the level of GSH were measured in compliance with previously optimized protocols.

For FRAP assay, 2,4,6-tris(2-pyridyl)-s-triazine (Sigma-Aldrich) was used as a chromogenic reagent. The assay comes from published protocols (Bordonaba and Terry, 2012; Rodriguez-Naranjo *et al.*, 2012). It was solved at 10 mmol/l concentration in 40 mmol/l HCl and with 20 mmol/l FeCl_3_ in the ratio 1:1. After that, 5 ml of the freshly prepared mixture was added into 25 ml of 0.1 mol/l acetate buffer pH 3.6 and kept at 37 °C for 10 minutes. 200 µl of the freshly prepared reagent was mixed with 30 µl of the sample, diluted with 770 µl of deionized water and incubated for 10 minutes. Finally, the mixture was centrifuged at 10,000×g for another 10 minutes and absorbance was measured at 593 nm. Molar concentration of FRAP value, i.e. molar value of reduced ferric atoms, was calculated using extinction coefficient ɛ=26,000 l/mol×cm.

TBARS assay was done in compliance with the published protocol (Granados-Oliveros *et al.*, 2013; Granot *et al.*, 1999; Luchtemberg *et al.*, 2008; Papandreou *et al.*, 2012; Wang *et al.*, 2011). First, 67 mg of thiobarbituric acid (Sigma-Aldrich) was solved in 1 ml of dimethylsulfoxide and 9 ml of deionized water shortly before assay. After that, 200 µl of the sample prepared in compliance with the previous protocol was poured into 400 µl of 10% trichloroacetic acid and incubated for 15 minutes in an ice bath and spun at 3,000×g for 15 minutes. Finally, 400 µl of the treated sample was added to 400 µl of the before described thiobarbituric acid solution and heated at 100 °C for 10 minutes. Absorbance at 532 nm was measured after cooling down of the mixture. The molar value of TBARS was calculated using the extinction coefficient for malondialdehyde-thiobarbituric acid complex ɛ=156,000 l/mol×cm.

GSH reacts with 5,5′dithiobis (2-nitrobenzoic acid), which can be used for its quantification. From the samples, high molecular weight peptides and proteins were removed by mixing 50 µl of tissue homogenate with 50 µl 2.5% (v/v) trichloroacetic acid. Finally, the precipitate was spun at 12,000 ×g for 5 minutes. The fresh supernatant was neutralized by adding 50 µl 0.25 mmol/l NaOH and mixed with 450 µl of 5,5′dithiobis (2-nitrobenzoic acid) 0.4 mg/ml (Sigma-Aldrich). Absorbance at 412 nm was measured one minute after preparation of the mixture. Molar concentration of GSH was calculated from the nitrothiobenzoate extinction coefficient ɛ=14,150 l/mol×cm.

GR oxidizes NADPH and the reaction can be used for GR activity measurement. In a disposable cuvette, 100 µl of 10 mmol/l of oxidized glutathione (Sigma-Aldrich) was poured with the same volume of 1 mmol/l NADPH (Sigma-Aldrich). Both reagents were solved in water. In the mixture, pH was adjusted to 7.4 by 650 µl of phosphate buffered saline addition. Finally, 100 µl of 10 mmol/l EDTA was injected, followed by the sample (50 µl). Absorbance at 340 nm was measured after 30 seconds and then after 150 seconds. GR activity was calculated from absorbance change using the extinction coefficient ɛ=6,220 l/mol×cm.

GST activity was assayed in compliance with the following protocol: 10 µl of 100 mmol/l 1-chloro-2,4-dinitrobezene was added to 10 µl of GSH. In the mixture, pH was stabilized using 980 µl of phosphate buffered saline. Reaction was initiated by addition of 50 µl tissue homogenate. Absorbance was measured at 340 nm 30 seconds after mixing and then after 150 seconds. Enzyme activity was calculated considering the extinction coefficient 9,600 l/mol×cm and measured absorbance shift.

### Statistical processing of experimental data

Origin 8 SR2 (OriginLab Corporation, Northampton, MA, USA) was used for statistical purposes. One way ANOVA with Scheffe test were chosen for significance testing. Probability level 0.05 was calculated for a group size eight specimens. The experimental data were expressed as mean ± standard deviation.

## Results and discussion

The laboratory mice had no manifestation typical of poisoning by AChE inhibitors, such as miosis, salivation, urination or convulsion (Bucaretchi *et al.*, 2012; Giadinis *et al.*, 2009; Pohanka, 2012a). Minor behavioral alterations were observed in the animals which received the upper dose of neostigmine. However, the alterations such as anxiety were not extensively researched and disappeared in hours after the exposure. The findings are not surprising as the neostigmine doses used were calculated from recommended therapeutic doses for humans, considering the weight of the animals. Neostigmine is used as a drug of choice for suppression of myasthenia gravis manifestations and in anesthesia for reversing the effect of non-depolarizing muscle relaxants (Dahaba *et al.*, 2012; Foy *et al.*, 2011; Haines & Thurtell, 2012; Johnson *et al.*, 2011). The median dose used in the experiment, 40.0 µg/kg, approximately corresponds to the human therapeutic dose (Lederer *et al.*, 2010). The upper dose used in the experiment, 200 µg/kg, was under the median lethal dose for mice, which is between 0.3–0.4 mg/kg (Starec *et al.*, 1997). It can be assumed that increase of the neostigmine dose of 200 µg/kg used in the experiment would be followed by extensive manifestation of poisoning. In this experiment, the decision to choose the therapeutic dose rather than a dose causing poisoning was motivated by the effort to make it easy to extrapolate the results to humans taking neostigmine as a drug.

The assays of AChE, caspase-3, GSH, GST, GR and TBARS did not show any significant alterations either in the liver or spleen. The experimental values of the markers are obvious from Table 1 for the liver and Table 2 for the spleen. For AChE, the results are in compliance with the expectation that neostigmine is a pseudo-irreversible inhibitor spontaneously decarbamylated from AChE′s active site and the activity of AChE is recovered (Burnell & Wilkins, 1988; Harris *et al.*, 1989; Pohanka, 2011b, 2012a). Three days after beginning of the experiment, full return of AChE activity can be expected and the experimental data confirm this as no alteration in AChE activity was revealed. The findings concerning the alterations in the other markers can be attributed to a long term lasting covering of an oxidative insult rather than to long lasting inhibition of the enzyme.

**Table 1:** Selected markers in liver.

Neostigmine (µg/kg)	0	8.00	40.0	200
AChE (nkat/g)	4.74±0.21	5.05±0.39	4.65±0.36	4.55±0.29
Caspase-3 (µkat/g)	19.5±2.7	21.1±3.4	19.7±4.2	22.6±1.8
GSH (µmol/g)	1.05±0.23	1.12±0.18	1.18±0.14	1.15±0.27
GST (nkat/g)	621±58	598±71	687±49	623±56
GR (nkat/g)	99.3±7.6	95.8±8.3	97.4±5.2	98.4±6.4
TBARS (nmol/g)	146±35	159±29	157±25	162±31

**Table 2:** Selected markers in spleen.

Neostigmine (µg/kg)	0	8.00	40.0	200
AChE (nkat/g)	1.72±0.32	1.91±0.41	1.67±0.28	1.84±0.25
Caspase-3 (µkat/g)	26.7±3.4	25.8±4.1	27.9±2.9	29.8±4.2
GSH (nmol/g)	955±59	967±42	959±75	974±69
GST (nkat/g)	47.7±5.4	52.5±4.6	49.3±7.2	53.7±4.8
GR (nkat/g)	85.4±3.7	84.9±2.5	86.7±5.2	87.1±3.4
TBARS (nmol/g)	104±15	112±21	117±18	109±24

Owing to the experimental data, no apoptotic processes occurred three days after exposure to neostigmine. This is not surprising as caspase-3 expression lasts for hours immediately after an apoptotic stimulus and disappears the following day (Ananth *et al.*, 2001; Tao *et al.*, 2006). Due to the cholinergic system, caspase-3 up-regulation can arise when acute toxicity appears in the course of AChE inhibition (RamaRao *et al.*, 2011). The finding reported here is in compliance with these expectations.

Steady activity of GR and the level of GSH with TBARS point to the fact that no oxidative insult remains over an interval of three days after poisoning. TBARS is a marker of lipid peroxidation indicating uncovered production of reactive oxygen species (Papandreou *et al.*, 2012). No increase in TBARS value can be interpreted that oxidative stress was not initiated in the body. GR reduces oxidized glutathione back to the GSH form. When an oxidative insult occurs, GR becomes expressed and returns the level of GSH (Yang *et al.*, 2012). Stable activity of GR and stable level of GSH can be interpreted as no serious oxidative insult following neostigmine application three days after poisoning. GST utilizes GSH for detoxification reaction (Duarte-Salles *et al.*, 2012). We can interpret the result that there is no specific demand for GSH and that GST is not involved in a long term effect of neostigmine.

**Figure 1:**
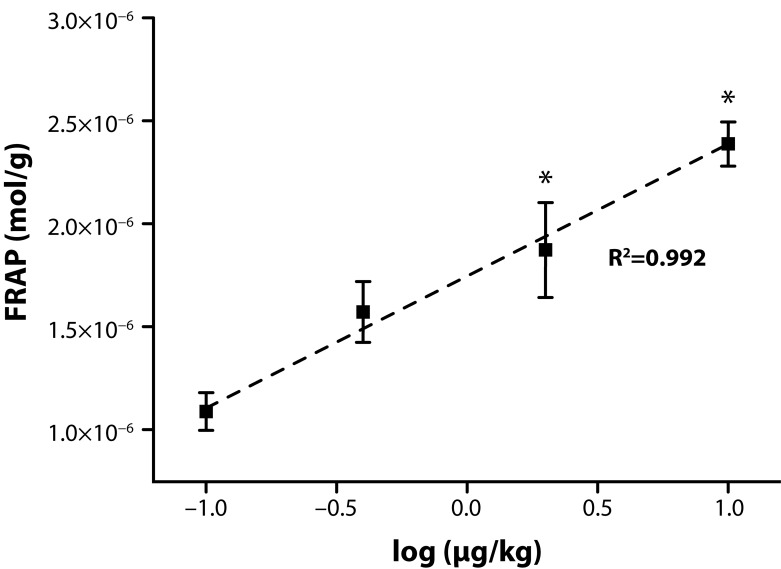
Ferric reducing antioxidant power of liver tissue. Error bars indicate standard deviation. Dose of neostigmine is mentioned on the x axis. Logarithmic value –1 on the x axis responds to controls receiving saline only.

**Figure 2:**
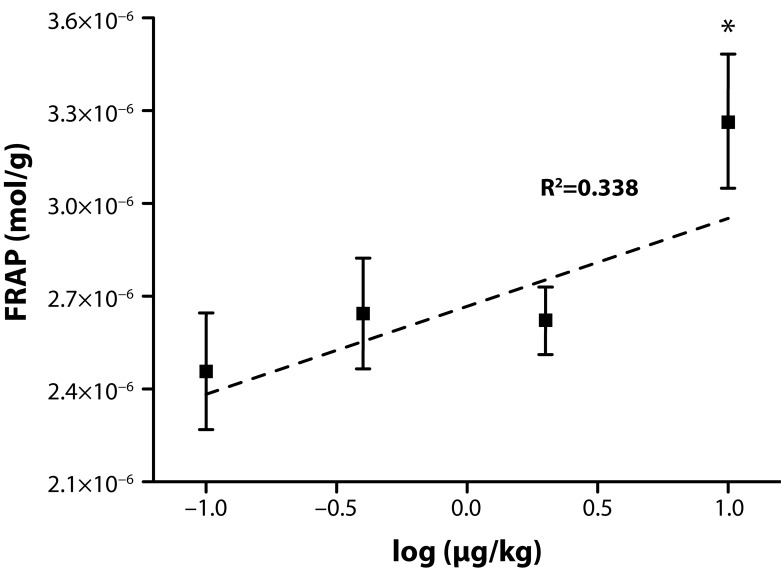
Ferric reducing antioxidant power of spleen tissue. Description is the same as in Figure 1.

A surprising phenomenon appeared when the FRAP value was determined. We revealed an accumulation of low molecular weight antioxidants in both the liver (Figure 1) and spleen (Figure 2). The value of FRAP was increased in the livers in a dose response manner and the increase was significant in the animals receiving neostigmine in the dose of 40.0 and 200 µg/kg. The dose response relation had a good regression coefficient of 0.992, indicating dependence of the low molecular weight antioxidants on the neostigmine dose, signaled by FRAP. In the spleen, the FRAP value was also increased. The increase was however significant only in animals which received the upper dose of neostigmine. The regression coefficient of FRAP in the spleen was lower than in the liver: 0.332. A higher resistance of livers to neostigmine can be claimed.

The data related to FRAP indicate an extensive increase in low molecular weight antioxidants; however, GSH is not involved in the process as its level is independent of the neostigmine dose. The increase was more extensive in the livers where nearly a 2.5 times increase of the FRAP value was found. On first appearance, the results might seem to neglect the idea that depletion of low antioxidants and generation or reactive oxygen and nitrogen species follow exposure to AChE inhibitors. Moreover, it is assumed that application of exogenous antioxidants can resolve some adverse effects caused by AChE inhibitors (Gupta *et al.*, 2001). Yet acute toxicity has to be distinguished from a long term effect. While antioxidants can become depleted in a short period after poisoning, their level becomes probably either re-constituted or even improved after manifestations of acute poisoning become resolved. Regulation of antioxidant metabolism based on the central nervous system can be neglected because neostigmine has a limited ability to cross the blood-brain barrier and has the ability to influence only peripheral nerves (Parisi & Francia, 2000).

It is noteworthy that AChE can become also expressed in the course of its inhibition or stress conditions (Darreh-Shori & Soininen, 2010; de Oliveira *et al.*, 2012). The improvement in the level of low molecular weight antioxidants reported here is beneficial for keeping the homeostasis of basic reactive oxygen species. It should be emphasized that neostigmine can affect the immune system, regulate inflammation and thus act also on antioxidants (Akinci *et al.*, 2005).

## Conclusions

Though neostigmine is well known as a stressogenic compound, the paper showed its ability to enhance accumulation of low molecular weight antioxidants in two organs studied, i.e. the liver and spleen. The accumulation is not related to GSH and its metabolisms as there was no plausible effect on the markers tested. We can conclude that antioxidant therapy by an AChE inhibitor can be effective when used early after poisoning. The body has its own mechanism to resolve depletion of antioxidants and improve oxidative homeostasis even without external intervention.
